# 1-(4-Benz­yloxy-5-meth­oxy-2-nitro­phen­yl)ethanone

**DOI:** 10.1107/S1600536809011532

**Published:** 2009-04-08

**Authors:** Lei Gao, Li-hua Guo, Rui Liu, Dan-hua Que, Hong-fei Ma

**Affiliations:** a Department of Applied Chemistry, College of Science, Nanjing University of Technology, Nanjing 210009, People’s Republic of China

## Abstract

In the mol­ecule of the title compound, C_16_H_15_NO_5_, the aromatic rings are oriented at a dihedral angle of 74.89 (3)°. Intra­molecular C—H⋯O inter­actions result in the formation of a seven-membered ring. In the crystal structure, weak inter­molecular C—H⋯O inter­actions link the mol­ecules into chains along the *b* axis.

## Related literature

The title compound is an important pharmaceutical intermediate. For general background, see: Mizuta *et al.* (2002 ▶). For bond-length data, see: Allen *et al.* (1987 ▶). 
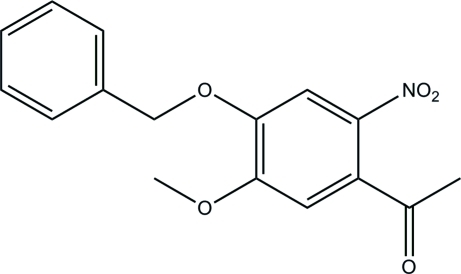


## Experimental

### 

#### Crystal data

C_16_H_15_NO_5_
*M* *_r_* = 301.29
Orthorhombic, 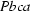

*a* = 13.390 (3) Å
*b* = 10.465 (2) Å
*c* = 20.768 (4) Å
*V* = 2910.1 (10) Å^3^
*Z* = 8
Mo *K*α radiation
μ = 0.10 mm^−1^
*T* = 294 K
0.20 × 0.10 × 0.10 mm

#### Data collection

Enraf–Nonius CAD-4 diffractometer
Absorption correction: ψ scan (North *et al.*, 1968 ▶) *T*_min_ = 0.980, *T*_max_ = 0.990
2634 measured reflections
2634 independent reflections
1446 reflections with *I* > 2σ(*I*)
3 standard reflections frequency: 120 min intensity decay: none

#### Refinement

*R*[*F* ^2^ > 2σ(*F* ^2^)] = 0.071
*wR*(*F* ^2^) = 0.223
*S* = 1.06
2634 reflections
199 parameters
H-atom parameters constrained
Δρ_max_ = 0.24 e Å^−3^
Δρ_min_ = −0.26 e Å^−3^

### 

Data collection: *CAD-4 Software* (Enraf–Nonius, 1989 ▶); cell refinement: *CAD-4 Software*; data reduction: *XCAD4* (Harms & Wocadlo, 1995 ▶); program(s) used to solve structure: *SHELXS97* (Sheldrick, 2008 ▶); program(s) used to refine structure: *SHELXL97* (Sheldrick, 2008 ▶); molecular graphics: *ORTEP-3 for Windows* (Farrugia, 1997 ▶) and *PLATON* (Spek, 2009 ▶); software used to prepare material for publication: *SHELXTL* (Sheldrick, 2008 ▶).

## Supplementary Material

Crystal structure: contains datablocks I, global. DOI: 10.1107/S1600536809011532/hk2654sup1.cif

Structure factors: contains datablocks I. DOI: 10.1107/S1600536809011532/hk2654Isup2.hkl

Additional supplementary materials:  crystallographic information; 3D view; checkCIF report

## Figures and Tables

**Table 1:** Hydrogen-bond geometry (Å, °)

*D*—H⋯*A*	*D*—H	H⋯*A*	*D*⋯*A*	*D*—H⋯*A*
C4—H4*A*⋯O3^i^	0.93	2.58	3.466 (5)	158
C16—H16*C*⋯O4	0.96	2.35	2.899 (5)	115

Symmetry code: (i) 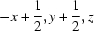.

**Fig. 1.**
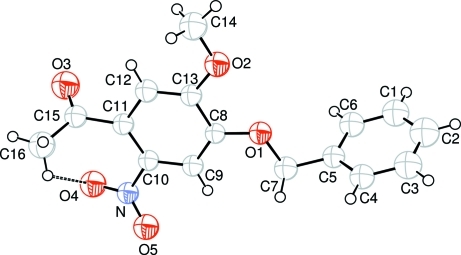
The molecular structure of the title molecule, with the atom-numbering scheme.

**Fig. 2.**
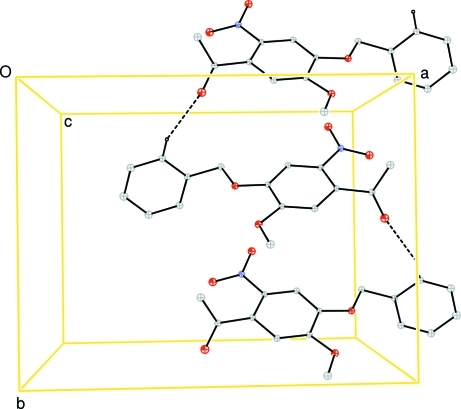
A partial packing diagram of the title compound. Hydrogen bonds are shown as dashed lines. Hydrogen atoms not involved in hydrogen bonding have been omitted.

## supplementary crystallographic information

### Comment

The title compound contains nitro, acetyl and methoxy groups, which can react with different groups to prepare various functional organic compounds as a fine organic intermediate. We report herein its crystal structure.

In the molecule of the title compound (Fig. 1), the bond lengths (Allen *et al.*,  1987) and angles are within normal ranges. Rings A (C1-C6) and B (C8-C13) are, of course, planar and they are oriented at a dihedral angle of 74.89 (3)°. Intramolecular C-H···O interaction (Table 1) results in the formation of a seven-membered ring C (O4/N/C10/C11/C15/C16/H16C) having twisted conformation.

In the crystal structure, weak intermolecular C-H···O interactions (Table 1) link the molecules into chains along the b axis (Fig. 2), in which they may be effective in the stabilization of the structure.

### Experimental

For the preparation of the title compound, 1-(4-benzyloxy-5-methoxy-2-nitro- phenyl)ethanone (20.0 g, 66.4 mmol) were dissolved in DMF (50 ml). Then, the solution was poured into ice water (100 ml). The crystalline product was isolated by filtration, washed with water (600 ml). Crystals suitable for X-ray analysis were obtained by slow evaporation of an ethanol solution.

### Refinement

H atoms were positioned geometrically, with C-H = 0.93, 0.97 and 0.96 Å for aromatic, methylene and methyl H, respectively, and constrained to ride on their parent atoms, with U_iso_(H) = xU_eq_(C), where x = 1.5 for methyl H and x = 1.2 for all other H atoms.

### Crystal data

**Table:**

C_16_H_15_NO_5_	*F*(000) = 1264
*M**_r_* = 301.29	*D*_x_ = 1.375 Mg m^−^^3^
Orthorhombic, *P**b**c**a*	Mo *K*α radiation, λ = 0.71073 Å
Hall symbol: -P 2ac 2ab	Cell parameters from 25 reflections
*a* = 13.390 (3) Å	θ = 1.0–1.0°
*b* = 10.465 (2) Å	µ = 0.10 mm^−^^1^
*c* = 20.768 (4) Å	*T* = 294 K
*V* = 2910.1 (10) Å^3^	Needle, colorless
*Z* = 8	0.20 × 0.10 × 0.10 mm

### Data collection

**Table:**

Enraf–Nonius CAD-4 diffractometer	1446 reflections with *I* > 2σ(*I*)
Radiation source: fine-focus sealed tube	*R*_int_ = 0.0000
graphite	θ_max_ = 25.2°, θ_min_ = 2.0°
ω/2θ scans	*h* = 0→16
Absorption correction: ψ scan (North *et al.*, 1968)	*k* = 0→12
*T*_min_ = 0.980, *T*_max_ = 0.990	*l* = 0→24
2634 measured reflections	3 standard reflections every 120 min
2634 independent reflections	intensity decay: none

### Refinement

**Table:**

Refinement on *F*^2^	Primary atom site location: structure-invariant direct methods
Least-squares matrix: full	Secondary atom site location: difference Fourier map
*R*[*F*^2^ > 2σ(*F*^2^)] = 0.071	Hydrogen site location: inferred from neighbouring sites
*wR*(*F*^2^) = 0.223	H-atom parameters constrained
*S* = 1.06	*w* = 1/[σ^2^(*F*_o_^2^) + (0.118*P*)^2^] where *P* = (*F*_o_^2^ + 2*F*_c_^2^)/3
2634 reflections	(Δ/σ)_max_ < 0.001
199 parameters	Δρ_max_ = 0.24 e Å^−^^3^
0 restraints	Δρ_min_ = −0.26 e Å^−^^3^

### Special details

**Table:**

Geometry. All e.s.d.'s (except the e.s.d. in the dihedral angle between two l.s. planes) are estimated using the full covariance matrix. The cell e.s.d.'s are taken into account individually in the estimation of e.s.d.'s in distances, angles and torsion angles; correlations between e.s.d.'s in cell parameters are only used when they are defined by crystal symmetry. An approximate (isotropic) treatment of cell e.s.d.'s is used for estimating e.s.d.'s involving l.s. planes.
Refinement. Refinement of *F*^2^ against ALL reflections. The weighted *R*-factor *wR* and goodness of fit *S* are based on *F*^2^, conventional *R*-factors *R* are based on *F*, with *F* set to zero for negative *F*^2^. The threshold expression of *F*^2^ > σ(*F*^2^) is used only for calculating *R*-factors(gt) *etc*. and is not relevant to the choice of reflections for refinement. *R*-factors based on *F*^2^ are statistically about twice as large as those based on *F*, and *R*- factors based on ALL data will be even larger.

### Fractional atomic coordinates and isotropic or equivalent isotropic displacement parameters (Å^2^)

**Table:**

	*x*	*y*	*z*	*U*_iso_*/*U*_eq_	
N	0.4084 (2)	0.3120 (3)	0.33235 (15)	0.0456 (8)	
O1	0.07225 (17)	0.1644 (3)	0.38239 (11)	0.0460 (7)	
O2	0.13768 (18)	0.0124 (3)	0.46870 (13)	0.0538 (8)	
O3	0.5275 (2)	0.0290 (3)	0.42219 (18)	0.0748 (10)	
O4	0.4987 (2)	0.2830 (3)	0.33126 (15)	0.0684 (10)	
O5	0.3740 (2)	0.4020 (3)	0.30338 (14)	0.0633 (9)	
C1	−0.1972 (3)	0.0558 (5)	0.2841 (2)	0.0683 (13)	
H1A	−0.2128	−0.0196	0.2626	0.082*	
C2	−0.2731 (4)	0.1305 (6)	0.3092 (3)	0.0793 (16)	
H2A	−0.3394	0.1054	0.3051	0.095*	
C3	−0.2488 (3)	0.2420 (5)	0.3400 (3)	0.0682 (13)	
H3A	−0.2994	0.2942	0.3558	0.082*	
C4	−0.1506 (3)	0.2778 (4)	0.3478 (2)	0.0546 (11)	
H4A	−0.1357	0.3519	0.3706	0.065*	
C5	−0.0736 (3)	0.2049 (4)	0.32217 (18)	0.0461 (10)	
C6	−0.0996 (3)	0.0917 (4)	0.2906 (2)	0.0592 (12)	
H6A	−0.0495	0.0399	0.2737	0.071*	
C7	0.0318 (3)	0.2420 (4)	0.3310 (2)	0.0510 (10)	
H7A	0.0690	0.2277	0.2915	0.061*	
H7B	0.0364	0.3318	0.3420	0.061*	
C8	0.1732 (3)	0.1672 (3)	0.39057 (17)	0.0380 (9)	
C9	0.2392 (3)	0.2446 (4)	0.35806 (18)	0.0428 (9)	
H9A	0.2159	0.3038	0.3282	0.051*	
C10	0.3402 (2)	0.2336 (4)	0.37010 (17)	0.0409 (9)	
C11	0.3807 (3)	0.1481 (4)	0.41517 (18)	0.0430 (9)	
C12	0.3098 (3)	0.0707 (4)	0.44838 (19)	0.0478 (10)	
H12A	0.3323	0.0118	0.4786	0.057*	
C13	0.2105 (3)	0.0807 (4)	0.43705 (18)	0.0422 (9)	
C14	0.1676 (3)	−0.0629 (5)	0.5236 (2)	0.0661 (13)	
H14A	0.1103	−0.1054	0.5413	0.099*	
H14B	0.1964	−0.0080	0.5557	0.099*	
H14C	0.2160	−0.1253	0.5104	0.099*	
C15	0.4875 (3)	0.1311 (4)	0.43162 (19)	0.0478 (10)	
C16	0.5384 (3)	0.2346 (5)	0.4663 (2)	0.0652 (13)	
H16A	0.6068	0.2115	0.4735	0.098*	
H16B	0.5059	0.2486	0.5069	0.098*	
H16C	0.5356	0.3115	0.4411	0.098*	

### Atomic displacement parameters (Å^2^)

**Table:**

	*U*^11^	*U*^22^	*U*^33^	*U*^12^	*U*^13^	*U*^23^
N	0.0501 (17)	0.049 (2)	0.0377 (18)	−0.0016 (16)	0.0013 (14)	−0.0030 (16)
O1	0.0448 (14)	0.0553 (18)	0.0378 (15)	−0.0030 (12)	−0.0076 (11)	0.0163 (13)
O2	0.0561 (15)	0.0561 (19)	0.0492 (16)	−0.0062 (13)	−0.0013 (13)	0.0211 (14)
O3	0.0644 (19)	0.056 (2)	0.104 (3)	0.0188 (17)	−0.0083 (17)	−0.0031 (19)
O4	0.0493 (16)	0.083 (2)	0.073 (2)	−0.0008 (15)	0.0050 (14)	0.0105 (18)
O5	0.0683 (18)	0.057 (2)	0.065 (2)	−0.0072 (15)	−0.0065 (15)	0.0197 (17)
C1	0.076 (3)	0.059 (3)	0.070 (3)	−0.014 (3)	−0.017 (3)	−0.009 (2)
C2	0.059 (3)	0.081 (4)	0.098 (4)	−0.018 (3)	−0.016 (3)	0.019 (3)
C3	0.049 (2)	0.067 (3)	0.089 (3)	0.004 (2)	0.003 (2)	0.009 (3)
C4	0.055 (2)	0.048 (3)	0.060 (3)	0.003 (2)	0.005 (2)	0.000 (2)
C5	0.055 (2)	0.041 (2)	0.043 (2)	0.0047 (18)	−0.0095 (17)	0.0119 (19)
C6	0.064 (3)	0.050 (3)	0.063 (3)	0.001 (2)	−0.009 (2)	−0.001 (2)
C7	0.053 (2)	0.054 (3)	0.046 (2)	0.001 (2)	−0.0072 (18)	0.009 (2)
C8	0.0472 (19)	0.037 (2)	0.0300 (18)	−0.0084 (16)	−0.0018 (15)	0.0034 (16)
C9	0.0491 (19)	0.037 (2)	0.042 (2)	0.0030 (17)	−0.0059 (17)	0.0082 (17)
C10	0.0400 (18)	0.043 (2)	0.039 (2)	−0.0042 (16)	−0.0018 (16)	−0.0059 (17)
C11	0.050 (2)	0.038 (2)	0.040 (2)	0.0024 (18)	−0.0055 (17)	0.0017 (18)
C12	0.053 (2)	0.040 (2)	0.050 (3)	0.0087 (18)	−0.0106 (19)	0.0086 (18)
C13	0.050 (2)	0.036 (2)	0.041 (2)	−0.0040 (17)	−0.0026 (17)	0.0077 (17)
C14	0.075 (3)	0.066 (3)	0.057 (3)	−0.010 (2)	−0.008 (2)	0.019 (2)
C15	0.050 (2)	0.052 (3)	0.041 (2)	0.0042 (19)	−0.0028 (17)	−0.002 (2)
C16	0.059 (2)	0.073 (3)	0.063 (3)	0.001 (2)	−0.016 (2)	−0.016 (3)

### Geometric parameters (Å, °)

**Table:**

N—O5	1.209 (4)	C6—H6A	0.9300
N—O4	1.247 (4)	C7—H7A	0.9700
N—C10	1.456 (5)	C7—H7B	0.9700
O1—C8	1.363 (4)	C8—C9	1.376 (5)
O1—C7	1.446 (4)	C8—C13	1.414 (5)
O2—C13	1.376 (4)	C9—C10	1.380 (5)
O2—C14	1.443 (5)	C9—H9A	0.9300
O3—C15	1.212 (5)	C10—C11	1.404 (5)
C1—C6	1.367 (5)	C11—C12	1.426 (5)
C1—C2	1.384 (7)	C11—C15	1.480 (5)
C1—H1A	0.9300	C12—C13	1.355 (5)
C2—C3	1.370 (7)	C12—H12A	0.9300
C2—H2A	0.9300	C14—H14A	0.9600
C3—C4	1.377 (5)	C14—H14B	0.9600
C3—H3A	0.9300	C14—H14C	0.9600
C4—C5	1.389 (5)	C15—C16	1.468 (6)
C4—H4A	0.9300	C16—H16A	0.9600
C5—C6	1.398 (6)	C16—H16B	0.9600
C5—C7	1.475 (5)	C16—H16C	0.9600
			
O5—N—O4	123.3 (3)	C9—C8—C13	119.0 (3)
O5—N—C10	117.9 (3)	C8—C9—C10	119.4 (3)
O4—N—C10	118.7 (3)	C8—C9—H9A	120.3
C8—O1—C7	116.8 (3)	C10—C9—H9A	120.3
C13—O2—C14	117.7 (3)	C9—C10—C11	123.5 (3)
C6—C1—C2	120.6 (5)	C9—C10—N	118.0 (3)
C6—C1—H1A	119.7	C11—C10—N	118.4 (3)
C2—C1—H1A	119.7	C10—C11—C12	115.3 (3)
C3—C2—C1	118.9 (4)	C10—C11—C15	127.1 (3)
C3—C2—H2A	120.6	C12—C11—C15	117.6 (3)
C1—C2—H2A	120.6	C13—C12—C11	121.7 (4)
C2—C3—C4	120.9 (5)	C13—C12—H12A	119.1
C2—C3—H3A	119.5	C11—C12—H12A	119.1
C4—C3—H3A	119.5	C12—C13—O2	124.9 (4)
C3—C4—C5	120.9 (4)	C12—C13—C8	121.0 (3)
C3—C4—H4A	119.5	O2—C13—C8	114.1 (3)
C5—C4—H4A	119.5	O2—C14—H14A	109.5
C4—C5—C6	117.4 (4)	O2—C14—H14B	109.5
C4—C5—C7	121.2 (4)	H14A—C14—H14B	109.5
C6—C5—C7	121.3 (4)	O2—C14—H14C	109.5
C1—C6—C5	121.1 (4)	H14A—C14—H14C	109.5
C1—C6—H6A	119.4	H14B—C14—H14C	109.5
C5—C6—H6A	119.4	O3—C15—C16	121.6 (4)
O1—C7—C5	107.6 (3)	O3—C15—C11	119.7 (4)
O1—C7—H7A	110.2	C16—C15—C11	118.2 (4)
C5—C7—H7A	110.2	C15—C16—H16A	109.5
O1—C7—H7B	110.2	C15—C16—H16B	109.5
C5—C7—H7B	110.2	H16A—C16—H16B	109.5
H7A—C7—H7B	108.5	C15—C16—H16C	109.5
O1—C8—C9	126.0 (3)	H16A—C16—H16C	109.5
O1—C8—C13	115.0 (3)	H16B—C16—H16C	109.5
			
C6—C1—C2—C3	0.8 (8)	O4—N—C10—C11	14.6 (5)
C1—C2—C3—C4	−2.0 (8)	C9—C10—C11—C12	0.4 (5)
C2—C3—C4—C5	2.9 (7)	N—C10—C11—C12	−177.4 (3)
C3—C4—C5—C6	−2.5 (6)	C9—C10—C11—C15	−179.2 (4)
C3—C4—C5—C7	−179.4 (4)	N—C10—C11—C15	3.0 (6)
C2—C1—C6—C5	−0.5 (7)	C10—C11—C12—C13	−0.4 (6)
C4—C5—C6—C1	1.3 (6)	C15—C11—C12—C13	179.2 (4)
C7—C5—C6—C1	178.1 (4)	C11—C12—C13—O2	−177.7 (3)
C8—O1—C7—C5	168.6 (3)	C11—C12—C13—C8	1.3 (6)
C4—C5—C7—O1	100.4 (4)	C14—O2—C13—C12	8.6 (6)
C6—C5—C7—O1	−76.4 (5)	C14—O2—C13—C8	−170.5 (4)
C7—O1—C8—C9	5.7 (5)	O1—C8—C13—C12	177.8 (4)
C7—O1—C8—C13	−174.0 (3)	C9—C8—C13—C12	−2.0 (6)
O1—C8—C9—C10	−177.9 (3)	O1—C8—C13—O2	−3.1 (5)
C13—C8—C9—C10	1.9 (5)	C9—C8—C13—O2	177.1 (3)
C8—C9—C10—C11	−1.1 (6)	C10—C11—C15—O3	−118.1 (5)
C8—C9—C10—N	176.7 (3)	C12—C11—C15—O3	62.3 (5)
O5—N—C10—C9	16.4 (5)	C10—C11—C15—C16	69.2 (5)
O4—N—C10—C9	−163.3 (3)	C12—C11—C15—C16	−110.4 (4)
O5—N—C10—C11	−165.6 (3)		

### Hydrogen-bond geometry (Å, °)

**Table:**

*D*—H···*A*	*D*—H	H···*A*	*D*···*A*	*D*—H···*A*
C4—H4A···O3^i^	0.93	2.58	3.466 (5)	158
C16—H16C···O4	0.96	2.35	2.899 (5)	115

Symmetry codes: (i) −*x*+1/2, *y*+1/2, *z*.
